# Genomewide and Enzymatic Analysis Reveals Efficient d-Galacturonic Acid Metabolism in the Basidiomycete Yeast Rhodosporidium toruloides

**DOI:** 10.1128/mSystems.00389-19

**Published:** 2019-12-17

**Authors:** Ryan J. Protzko, Christina A. Hach, Samuel T. Coradetti, Magdalena A. Hackhofer, Sonja Magosch, Nils Thieme, Gina M. Geiselman, Adam P. Arkin, Jeffrey M. Skerker, John E. Dueber, J. Philipp Benz

**Affiliations:** a Department of Molecular and Cell Biology, University of California, Berkeley, California, USA; b Energy Biosciences Institute, Berkeley, California, USA; c Holzforschung München, TUM School of Life Sciences Weihenstephan, Technische Universität München, Freising, Germany; d Environmental Genomics and Systems Biology Division, Lawrence Berkeley National Laboratory, Berkeley, California, USA; e Biological Systems & Engineering Division, Lawrence Berkeley National Laboratory, Berkeley, California, USA; f Department of Bioengineering, University of California, Berkeley, California, USA; University of Queensland

**Keywords:** *Rhodosporidium toruloides*, aerobic catabolism, carbon metabolism, galacturonic acid, yeasts

## Abstract

Biorefining of renewable feedstocks is one of the most promising routes to replace fossil-based products. Since many common fermentation hosts, such as Saccharomyces cerevisiae, are naturally unable to convert many component plant cell wall polysaccharides, the identification of organisms with broad catabolism capabilities represents an opportunity to expand the range of substrates used in fermentation biorefinery approaches. The red basidiomycete yeast Rhodosporidium toruloides is a promising and robust host for lipid- and terpene-derived chemicals. Previous studies demonstrated assimilation of a range of substrates, from C_5_/C_6_ sugars to aromatic molecules similar to lignin monomers. In the current study, we analyzed the potential of R. toruloides to assimilate d-galacturonic acid, a major sugar in many pectin-rich agricultural waste streams, including sugar beet pulp and citrus peels. d-Galacturonic acid is not a preferred substrate for many fungi, but its metabolism was found to be on par with those of d-glucose and d-xylose in R. toruloides. A genomewide analysis by combined transcriptome sequencing (RNA-seq) and RB-TDNA-seq revealed those genes with high relevance for fitness on d-galacturonic acid. While R. toruloides was found to utilize the nonphosphorylative catabolic pathway known from ascomycetes, the maximal velocities of several enzymes exceeded those previously reported. In addition, an efficient downstream glycerol catabolism and a novel transcription factor were found to be important for d-galacturonic acid utilization. These results set the basis for use of R. toruloides as a potential host for pectin-rich waste conversions and demonstrate its suitability as a model for metabolic studies with basidiomycetes.

**IMPORTANCE** The switch from the traditional fossil-based industry to a green and sustainable bioeconomy demands the complete utilization of renewable feedstocks. Many currently used bioconversion hosts are unable to utilize major components of plant biomass, warranting the identification of microorganisms with broader catabolic capacity and characterization of their unique biochemical pathways. d-Galacturonic acid is a plant component of bioconversion interest and is the major backbone sugar of pectin, a plant cell wall polysaccharide abundant in soft and young plant tissues. The red basidiomycete and oleaginous yeast Rhodosporidium toruloides has been previously shown to utilize a range of sugars and aromatic molecules. Using state-of-the-art functional genomic methods and physiological and biochemical assays, we elucidated the molecular basis underlying the efficient metabolism of d-galacturonic acid. This study identified an efficient pathway for uronic acid conversion to guide future engineering efforts and represents the first detailed metabolic analysis of pectin metabolism in a basidiomycete fungus.

## Abstract (precis)

The switch from the traditional fossil-based industry to a green and sustainable bioeconomy demands the complete utilization of renewable feedstocks. Many currently used bioconversion hosts are unable to utilize major components of plant biomass, warranting the identification of microorganisms with broader catabolic capacity and characterization of their unique biochemical pathways. d-Galacturonic acid is a plant component of bioconversion interest and is the major backbone sugar of pectin, a plant cell wall polysaccharide abundant in soft and young plant tissues. The red basidiomycete and oleaginous yeast Rhodosporidium toruloides has been previously shown to utilize a range of sugars and aromatic molecules. Using state-of-the-art functional genomic methods and physiological and biochemical assays, we elucidated the molecular basis underlying the efficient metabolism of d-galacturonic acid. This study identified an efficient pathway for uronic acid conversion to guide future engineering efforts and represents the first detailed metabolic analysis of pectin metabolism in a basidiomycete fungus.

## INTRODUCTION

Negative environmental impacts from fossil fuel consumption and volatile energy costs have accelerated academic and industrial efforts to develop sustainable commodity chemicals and biofuels via microbial fermentation of renewable plant biomass. Pectin-rich side streams from industrial processing of fruits and vegetables have a strong potential as fermentation feedstocks, as they are stably produced in high quantities and can be provided at low cost. Moreover, they accumulate centrally at their respective processing plants (reducing transport costs), are partly pretreated during processing, and are naturally devoid of lignin, overcoming major bottlenecks in lignocellulosic feedstock depolymerization. Furthermore, second-generation energy crops, such as agave and sugar beet, have high levels of pectin, sometimes exceeding 40% of the dry weight (1, 2). Despite these major advantages, pectin-rich feedstocks are largely disposed of in landfills and biogas plants or are sold as an inexpensive livestock feed after an energy-intensive drying and pelleting process. Utilizing these waste streams for the biorefinery would benefit the bioeconomy without augmenting current land use and decrease the contribution of these agricultural wastes to landfill overflow and environmental pollution through airborne spores from molds, which thrive on pectin-rich waste (3, 4).

Pectin is the most heterogeneous of the major plant cell wall polysaccharides and has four main structural classes: homogalacturononan (HG), rhamnogalacturonan I (RG-I), and the substituted HGs rhamnogalacturonan II (RG-II) and xylogalacturonan (XG). α-(1,4)-Linked d-galacturonic acid (d-galUA) is the major backbone sugar of all HG structures and can comprise up to 70% of the polysaccharide. d-galUA is a uronic sugar with the same hydroxyl configuration as d-galactose, but with a carboxylic acid group at the C-6 position. Other pectic monosaccharides include l-arabinose (l-ara), d-galactose (d-gal), l-rhamnose (l-rha), and d-xylose (d-xyl) (5, 6).

The catabolic pathway for d-galUA utilization has not yet been characterized in the Basidiomycota phylum. In ascomycetes, d-galUA is taken up by a major facilitator superfamily (MFS)-type transporter specific for uronic acids (7) and in a first step is reduced to l-galactonate by a d-galUA reductase, which either is NADPH specific or accepts either NADH or NADPH, depending on the organism (8, 9). Next, l-galactonate is transformed into 3-deoxy-l-threo-hex-2-ulosonate by a dehydratase (10) and then into l-glyceraldehyde and pyruvate by an aldolase (11). The last step of the reaction requires NADPH as a cofactor and is catalyzed by a glyceraldehyde reductase which converts l-glyceraldehyde to glycerol, a central metabolite (12).

Rhodosporidium toruloides is a strong candidate for bioconversion of pectin-rich waste streams. This basidiomycetous red yeast has been isolated from a wide variety of pectin-rich substrates (e.g., oranges [13], grapes, olives [14], and sugar beet pulp [14, 15]). R. toruloides can grow well on d-galUA as a sole carbon source (16), indicating an efficient pathway for d-galUA metabolism. Furthermore, R. toruloides is of increasing biotechnological interest as a host for bioconversions. The yeast naturally accumulates lipids and carotenoids, suggesting that it may be a promising host for the production of terpene- and lipid-based bioproducts (17). Additionally, the yeast can coutilize both hexose and pentose sugars (18, 19) and assimilate aromatic compounds, such as *p*-coumarate, derived from acylated lignins (20), suggesting advantages for efficient carbon utilization over conventional lignocellulosic conversion hosts. Finally, R. toruloides has advantages as a model system for basidiomycetes, as it is easily manipulated in laboratory settings, whereas the vast majority of known basidiomycetes are difficult to cultivate (21). Furthermore, genetic analyses and mutant strain development are becoming more efficient in R. toruloides as novel molecular tools are being developed (22–25).

The aim of the present study was to characterize the d-galUA utilization pathway of R. toruloides. Growth assays demonstrate that this pathway is highly efficient in comparison to the utilization of d-xyl or even d-glucose (d-glc). To identify all genes involved in d-galUA catabolism, parallel transcriptome sequencing (RNA-seq) and whole-genome RB-TDNA-seq studies were performed (22). The enzymes for each metabolic step were subsequently heterologously expressed in Escherichia coli and purified to verify their kinetic properties *in vitro*. Furthermore, we identified transporters and a novel transcription factor essential for d-galUA utilization. Finally, global carbon utilization trends underlying the high efficiency of d-galUA catabolism are discussed here. We believe that the results from this study offer crucial insights into basidiomycete d-galUA utilization and provide a starting point for engineering of R. toruloides as a host for pectin-rich waste bioconversion.

## RESULTS

### R. toruloides IFO0880 has a highly efficient d-galUA catabolism and can coutilize d-galUA with d-glucose and d-xylose.

**FIG 1:**
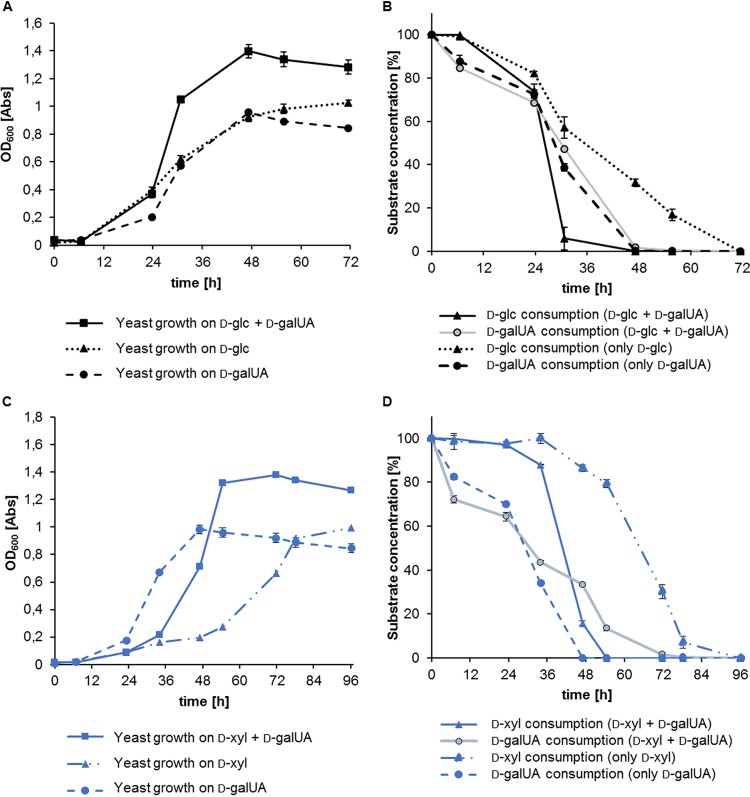
Growth assays of R. toruloides IFO0880 on single-carbon-source media exhibit efficient utilization of d-galUA compared to d-glc and d-xyl. Growth assays on mixed-carbon-source media show coconsumption of d-galUA with d-glu and d-galUA with d-xyl. (A) Culture growth (OD) over time on 50 mM (each) d-galUA, d-glc, and 1:1 mixed substrates. (B) Normalized sugar consumption of the same cultures. Dotted lines, dashed lines, and solid lines indicate d-glc, d-galUA, and mixed cultures, respectively. For sugar consumption of mixed cultures, d-glc consumption is shown by a black line, whereas d-galUA consumption is shown in gray. (C) Culture growth (OD) over time on 50 mM (each) d-galUA, d-xyl, and 1:1 mixed substrate. (D) Normalized sugar consumption of the same cultures. Dotted lines, dashed lines, and solid lines indicate d-xyl, d-galUA, and mixed cultures, respectively. For sugar consumption of mixed cultures, d-xyl consumption is shown as a blue line, whereas d-galUA consumption is shown in gray. Values are the means of three biological replicates. Error bars indicate SD.

Since it was known that R. toruloides can utilize both d-glc and d-xyl (18), we tested how the assimilation of d-galUA would compare to these rates and whether growth inhibition would be visible in mixed-substrate cultures. To this end, R. toruloides IFO0880 was grown in 200-μl-volume cultures with 50 mM (each) concentrations of these sugars as the sole carbon source as well as in cultures in which d-galUA was mixed with either d-glc or d-xyl in a 1:1 ratio. Surprisingly, despite a slightly slower acceleration phase (meaning the growth period between the lag phase [here approximately the first 6 h] and exponential growth phase [here after ∼24 h]) on d-galUA compared to d-glc in the first 24 h, culture densities of R. toruloides reached almost similar final optical densities (ODs) (Fig. 1A; see also Fig. S1A in the supplemental material). Moreover, d-galUA was completely consumed by that time, while total consumption of d-glc required about 70 h (Fig. 1B). With this rate, growth on d-galUA was faster than on d-xyl as the sole carbon source, which required about 80 h to reach the same density and more than 90 h to be completely consumed (Fig. 1C and D and Fig. S1B). In mixed cultures of d-galUA and d-glc, d-glc consumption was accelerated compared to that with single inoculations, indicating coutilization of the two sugars (Fig. 1B). The same was true for the cocultures of d-galUA and d-xyl (Fig. 1D and Fig. S1B). Also in this case, the presence of d-galUA led to an acceleration of d-xyl assimilation, while the d-galUA utilization was slightly delayed compared to that with the single inoculations.

10.1128/mSystems.00389-19.1 FIG S1 Growth assays of R. toruloides IFO0880 on either single- or mixed-carbon-source media (see Fig. 1) with OD_600_ values plotted in log_10_ scale over time. (A) Culture growth on 50 mM d-glc or d-galUA or mixed sugars with equal concentrations. The solid line indicates mixed-sugar conditions, the dotted line illustrates yeast growth on d-glc, and the dashed line shows growth on d-galUA. (B) Culture growth on 50 mM d-xyl or d-galUA or the two sugars mixed in equal concentrations. In blue, the solid line shows growth on both d-xyl and d-galUA media, whereas the dashed line illustrates growth on d-galUA as the sole carbon source. Growth on only d-xyl is shown as a dotted/dashed line. Download FIG S1, PDF file, 0.05 MB. Copyright © 2019 Protzko et al. 2019 Protzko et al. This content is distributed under the terms of the Creative Commons Attribution 4.0 International license.

**FIG 2:**
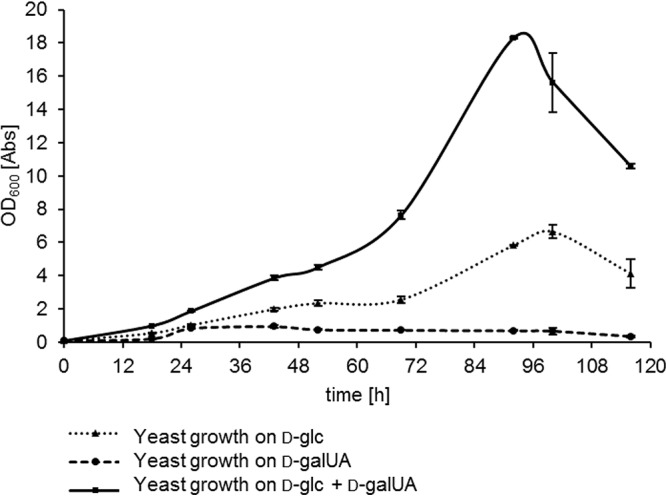
Growth assays of R. toruloides IFO0880 on single and mixed cultures (1:1) of d-glc and d-galUA on 500 mM substrate concentration in 50-ml culture volumes. Culture growth (OD) was measured over time. Dotted lines, dashed lines, and solid lines indicate d-glc, d-galUA, and mixed cultures, respectively. Values are the means of three biological replicates. Error bars indicate SD.

Since the above-described experiments were performed at low concentrations of monosaccharides, we performed an additional growth assay at 500 mM of substrate and a larger volume (50 ml) to resemble industrial settings with improved economics (Fig. 2). The high sugar loadings were tolerated by R. toruloides, reaching similar ODs like observed in the small cultures for d-galUA and about doubled culture densities on d-glc as the sole carbon source. The mixed-sugar condition led to an accelerated growth rate, corroborating the positive effect of coconsumption that was already visible in the initial assays. These results demonstrate that the presence of d-galUA appears not to be inhibitory to the catabolism of C_5_ and C_6_ sugars but rather leads to enhanced utilization.

### Identification of putative d-galUA utilization genes using differential RNA-seq analysis.

**FIG 3:**
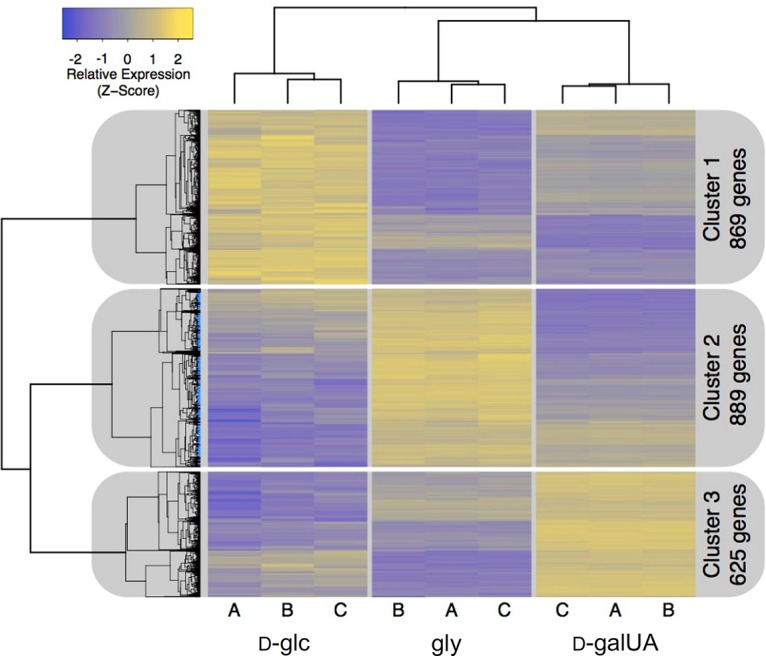
Hierarchical clustering of R. toruloides gene expression data reveals three major clusters based on carbon source. Cluster 1 contains 869 genes most highly expressed on d-glc, cluster 2 contains 889 genes most highly expressed on gly, and cluster 3 contains 625 genes most highly expressed on d-galUA.

We hypothesized that the genes involved in d-galUA utilization in R. toruloides could be identified by analyzing the transcriptional response to media containing d-galUA as the sole carbon source compared to media containing either d-glc or glycerol. Therefore, after growth of R. toruloides IFO0880 on either 2% d-galUA, 2% glycerol, or 2% d-glc, RNA was extracted during the log growth phase and the transcriptome analyzed by RNA-seq. Overall, more than 2,000 genes displayed differential transcript abundances between these three conditions, reflecting the significantly different requirements for growth on these carbon sources (Table S1). Hierarchical clustering separated the differentially expressed genes into three clusters of 869 genes most highly expressed on d-glc, 889 genes most highly expressed on glycerol, and 625 genes most highly expressed on d-galUA (Fig. 3). The last cluster included several genes with sequence similarity to genes for known enzymes participating in d-galUA catabolism in Aspergillus niger, Trichoderma reesei, and Neurospora crassa (Table 1).

**TABLE 1:** Putative enzymes involved in d-galUA catabolism are upregulated when R. toruloides is grown in d-galUA as a sole carbon source (Fig. 3, cluster 3)^*a*^

Protein ID	Homolog(s)	Reported/putative function(s)	FPKM		
			d-galUA	d-glc	gly
RTO4_12062	A. niger GaaB, T. reesei Lgd1, N. crassa NCU07064	l-Galactonic acid dehydratase	9,846	26	57
RTO4_11882	T. reesei Gar1	d-galUA reductase	7,667	118	64
RTO4_12061	A. niger GaaC, T. reesei Lga1, N. crassa NCU09532	2-Keto-3-deoxy-l-galactonate aldolase	6,397	24	134
RTO4_9774	A. niger GaaD (LarA, Alr, Err1), T. reesei Gld1	l-Glyceraldehyde reductase, pentose reductase, erythrose reductase	3,981	524	699

a The RTO4 protein identifier (ID), homologs to filamentous fungus d-galUA catabolic pathway genes, reported/putative function, and FPKM values are shown for d-galUA, d-glc, and gly.

10.1128/mSystems.00389-19.5 TABLE S1 Summary of RNA-seq and fitness data on d-galUA, d-glc, and glycerol. Average FPKM, fitness scores, and T statistics across three biological replicates for every gene in the R. toruloides IFO 0880 genome are shown. Genes are listed by their protein ID in version 4 of the genome publicly available at genome.jgi.doe.gov/Rhoto_IFO0880_4/. Orthologous genes in the S. cerevisiae genome are listed where clear orthologs exist. FPKM were tabulated with HISAT2 and Stringtie. Q-values (multiple hypothesis corrected *P* value) across all three carbon sources with the Ballgown package for the R statistical computing platform are included as a measure of statistically significant differential expression. Fitness scores are log_2_ ratios of barcode abundance after growth for 5 to 7 generations under the experimental condition versus the mutant pool before growth in the experimental condition. T-statstics are a measure consistency between different barcoded insertions in the same gene (see PMID 29521624 for details). Genes with |T| of >3 are considered to have significant fitness effects. The number of individual barcoded insertions with sufficient sequencing depth for fitness analysis is listed for each gene. Download Table S1, XLSX file, 1.2 MB. Copyright © 2019 Protzko et al. 2019 Protzko et al. This content is distributed under the terms of the Creative Commons Attribution 4.0 International license.

### Identification of genes required for d-galUA metabolism using genomewide fitness profiling.

**FIG 4:**
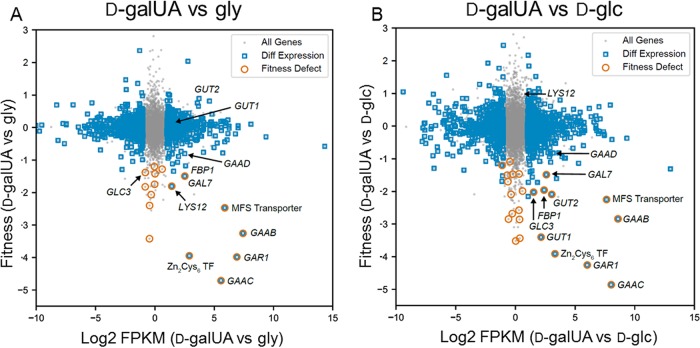
Plotting relative fitness scores versus differential expression of d-galUA grown R. toruloides identifies genes with essential function in d-galUA utilization. Genes with significant differential expression had a minimum FPKM of >5, at least a 2-fold difference in average FPKM between the two plotted conditions, and a multiple-hypothesis-adjusted *P* value of <0.05, as calculated across d-galUA), gly, and d-glc with the Ballgown analysis package for R. Genes with a relative fitness defect had relative T-statistics of less than −3 between conditions and relative fitness scores of less than −1 between the two plotted conditions. (A) Relative fitness scores versus relative transcript abundance for d-galUA versus gly grown cells. Catabolic pathway genes homologous to those in the A. niger and T. reesei utilization pathways (the GAR1, GaaB, GaaC, and GaaD genes), an MFS sugar transporter, and zinc finger transcription factor are clearly induced and required for fitness on d-galUA. (B) Relative fitness scores versus relative transcript abundance for d-galUA- versus d-glc-grown cells, illustrating additional pathways involved in d-galUA metabolism. Glycerol catabolism genes, such as the GUT1 and GUT2 genes, are found to be induced and required for fitness when cells are grown on d-galUA rather than d-glc.

To rapidly assess which R. toruloides genes are necessary for growth in d-galUA, we grew a sequence-barcoded random insertion library of R. toruloides IFO0880 on either 2% d-galUA, 2% gly, or 2% d-glc, similar to the case with the RNA-seq analysis described above. Insertions in genes necessary for growth in the respective carbon sources should prevent or slow growth in those conditions, thus leading to a depletion in the relative abundance of the sequence barcodes associated with those insertions (22). Transfer DNA (T-DNA) insertions in 28 genes led to significant growth defects on d-galUA versus d-glc, and insertions in 20 genes led to significant growth defects on d-galUA versus glycerol (Table 2 and Fig. 4). After filtering for statistical significance, we combined our RNA-seq data and fitness profiling data sets to gain further insight into the metabolism of d-galUA. Only seven genes had at least a 2-fold increase in transcript abundance on d-galUA and at least a 2-fold decrease in abundance for insertional mutants on d-galUA compared to glycerol and d-glc (Table 2). These genes included homologs to the previously characterized d-galUA utilization pathways in A. niger (GaaB, GaaC, and GaaD [9]) and Trichoderma reesei (GAR1 [8]). RTO4_9841, an MFS-type transporter related to pentose transporters (e.g., LAT-1 in N. crassa; NCU02188) was also both transcriptionally induced and required for robust growth on d-galUA, although other transporters were also specifically induced on d-galUA and may therefore be involved in the transport of d-galUA (Table 2). RTO4_13270, a fungus-specific zinc binuclear cluster transcription factor (TF), was also induced by d-galUA, and insertional mutants were severely deficient for growth on d-galUA, suggesting a primary role in regulating expression of d-galUA utilization enzymes. Finally, an ortholog of GAL7 was also induced and required for robust growth on d-galUA.

**TABLE 2:** Genes with d-galUA-specific induction and importance for fitness on d-galUA compared to gly and d-glc identify a hexose transporter, homologs to the ascomycete d-galUA catabolism pathway, and a putative d-galUA transcription factor

Protein ID	Name	Description	Mean FPKM			Fitness score		
			d-galUA	gly	d-glc	d-galUA	gly	d-glc
RTO4_9841		Hexose transporter	4,605	78	23	−2.3	0.2	−0.1
RTO4_11882	GAR1	Reductase	7,667	64	118	−4.0	0.0	0.3
RTO4_12062	GaaB	l-Galactonic acid dehydratase	9,846	57	26	−3.3	0.0	−0.4
RTO4_12061	GaaC	2-keto-3-deoxy-l-galactonate aldolase	6,397	134	24	−5.1	−0.4	−0.3
RTO4_9774	GaaD	NADPH-dependent erythrose reductase	3,981	699	524	−0.8	0.0	0.1
RTO4_11332	GAL7	Galactose-1-P uridyl transferase	330	58	55	−1.6	−0.1	−0.1
RTO4_13270		ZnCys transcription factor	93	12	9	−4.0	0.0	0.0

Additional genes were identified to be required for utilization of d-galUA and glycerol over d-glc (Table S2). These genes include members of the canonical glycerol utilization pathway, GUT1 and GUT2, and the glycerol proton symporter, STL1, the last showing a modest, but statistically significant, growth defect on glycerol. Mutants in homologs to members of the known carbon catabolite-regulating AMPK/SNF1 protein kinase complex (a Mig1/CreA/CRE-1 repressor; SNF1, SNF4, and SIP2) (26) were also deficient for growth on one or both of these alternative carbon sources, as were mutants in two G proteins (orthologs of S. cerevisiae CDC42 and Homo sapiens RAB6A) and likely interacting guanine exchange factors. Disruptions in thiolation of some tRNA residues also consistently resulted in small, but significant, fitness defects on d-galUA and glycerol but not on d-glc, further evidence that this process plays a role in nutrient sensing and carbon metabolism in diverse fungi (27, 28).

10.1128/mSystems.00389-19.6 TABLE S2 Genes from potentially interesting functional groups with fitness defects and/or induction on d-galUA and gly compared to d-glc identify global genetic factors required for respective carbon source utilization. Essential genes do not have fitness scores (N/A), as they are absent from the insertion library. FPKM values are shaded orange in proportion to expression level. Fitness scores are shaded blue in proportion to negative scores and orange for positive scores. Download Table S2, PDF file, 0.1 MB. Copyright © 2019 Protzko et al. 2019 Protzko et al. This content is distributed under the terms of the Creative Commons Attribution 4.0 International license.

### *In vitro* enzymatic characterization of the d-galUA catabolic proteins.

**FIG 5:**
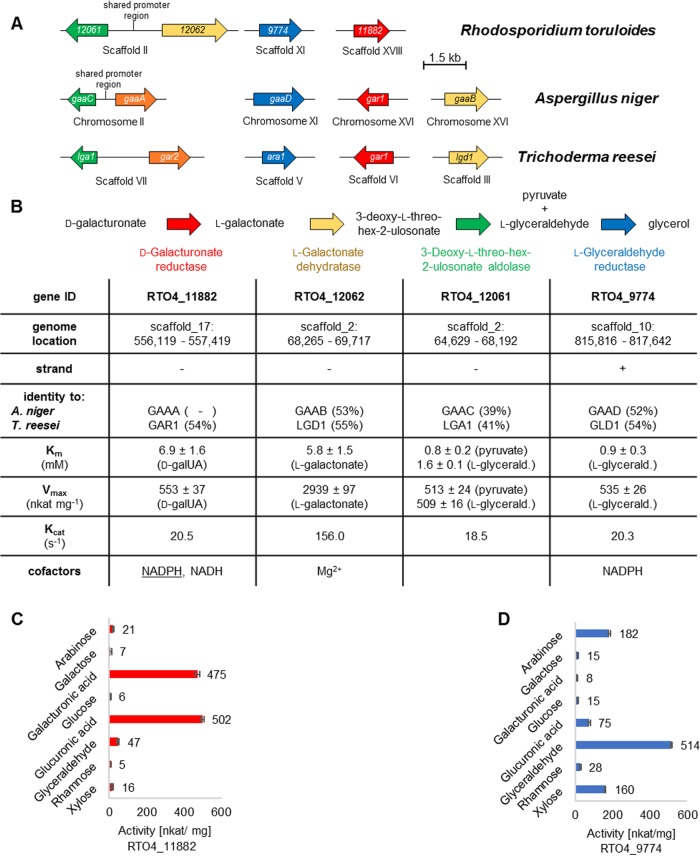
Gene arrangement and kinetic properties of the putative d-galUA-metabolizing enzymes in R. toruloides. (A) Gene synteny in comparison to A. niger and T. reesei. In R. toruloides, two genes appear linked in the genome, including a shared promoter region, but in contrast to the ascomycetes, it is those coding for the second and third steps of the pathway and not the first and third. (B) Table summarizing the major genomic and kinetic properties of the putative d-galUA-catabolizing genes and enzymes, respectively. Underlined NADPH indicates the favored cofactor of RTO4_11882. (C) Substrate spectrum of the putative d-galUA reductase. (D) Substrate spectrum of the putative l-glyceraldehyde reductase.

Based on the data described above and previous knowledge from ascomycetes, a model of d-galUA catabolism was hypothesized (Fig. 5). Intriguingly, a two-gene cluster was observed in R. toruloides, similar to what was described for ascomycetes (9). However, in this case, the *gaaC* homolog RTO4_12061 was linked not with the *gaaA* homolog (which is absent from the genome) but with RTO4_12062, the homolog of *gaaB* and *lgd1* in A. niger and T. reesei, respectively (Fig. 5A).

To confirm the corresponding enzyme activities, *in vitro* biochemical studies were performed. The enzymes were heterologously expressed in E. coli and purified to characterize their activity. The putative d-galUA reductase and GAR1 homolog RTO4_11882 displayed clear d-galUA reduction activity with a *K_m_* of about 7 mM (Fig. 5B and Fig. S2A). The *V*_max_ at saturating d-galUA concentrations was found to be 553 nkat/mg. A substrate scan revealed similarly high activities for this enzyme also on glucuronic acid, with only side activities on all other monosaccharides tested (Fig. 5C), suggesting that RTO4_11882 represents a uronic acid reductase. In addition, the enzyme was found to prefer NADPH as a cofactor and showed much weaker activity with NADH (Fig. S3). Dehydration of l-galactonate by the putative l-galactonate dehydratase (RTO4_12062) was observed at a high *V*_max_, 2,939 nkat/mg, with a *K_m_* of 5.8 mM (Fig. 5B and Fig. S2B) (29). Activity of the putative 3-deoxy-l-threo-hex-2-ulosonate aldolase (RTO4_12061) was tested by monitoring the reverse reaction of lglyceraldehyde and pyruvate (Fig. 5B and Fig. S2C). Affinities and velocities for both substrates were found to be very similar, with a *K_m_* in the range of about 1 mM and a *V*_max_ of about 510 nkat/mg (Fig. 5B). The putative l-glyceraldehyde reductase (RTO4_9774) displayed Michaelis-Menten kinetics of 0.9 mM, with a *V*_max_ of 535 nkat/mg, for l-glyceraldehyde (Fig. 5B and Fig. S2D). A substrate scan interestingly revealed that this enzyme also appears to be a major pentose reductase of R. toruloides, since robust activities were found for l-ara and d-xyl, with *K_m_*s in the range of 20 to 35 mM (Fig. 5D and Fig. S2D). Lower activities were recorded for other sugars, such as the hexoses d-glc and d-gal, the deoxy-hexose d-rha, and the uronic acid d-glucuronic acid.

10.1128/mSystems.00389-19.2 FIG S2 Protein purification and Michaelis-Menten kinetics of the recombinant d-galUA catabolism pathway enzymes. His-tagged proteins were expressed in E. coli and purified via IMAC; expected molecular weight and purity were determined by SDS-PAGE. Lanes: M, 2- to 212-kDa broad-range protein ladder (New England BioLabs [NEB], Germany) L, load; F, flow; W, wash; T, TEV digest; E, elutions. *In vitro* enzymatic assays were performed to determine the Michaelis-Menten kinetics of the enzymes. (A) The activity of RTO4_11882 with an expected protein size of 37.1 kDa was assessed by measuring the loss of NADPH over time. (B) Recombinant RTO4_12062 has an expected protein size of 55.7 kDa (His tagged) or 53.1 kDa (untagged after TEV cleavage). Activity was determined by a semicarbazide assay. (C) RTO4_12061 has a predicted size of 36.4 kDa, and the *in vitro* activity was determined in the reverse direction with the thiobarbiturate (TBA) assay. The two substrates were l-glyceraldehyde and pyruvate. (D) For RTO4_9774, with an expected protein size of 38.0 kDa, the NADPH loss during reduction of l-glyceraldehyde, l-ara, and d-xyl was measured. Data points are the means of triplicate measurements. Kinetics data were plotted with the IC50 Tool Kit (http://www.ic50.tk/). Download FIG S2, PDF file, 0.2 MB. Copyright © 2019 Protzko et al. 2019 Protzko et al. This content is distributed under the terms of the Creative Commons Attribution 4.0 International license.

10.1128/mSystems.00389-19.3 FIG S3 Enzyme activity of d-galUA reductase (RTO4_11882) using d-galUA, dxyl, or d-ara as a substrate (50 mM each) and either NADPH or NADH as a cofactor (0.3 mM). RTO4_11882 showed lower activity with NADH used as a cofactor than with NADPH. However, 8 to 21% of activity remained for NADH, depending on the substrate. Download FIG S3, PDF file, 0.02 MB. Copyright © 2019 Protzko et al. 2019 Protzko et al. This content is distributed under the terms of the Creative Commons Attribution 4.0 International license.

### Sugar reductase activities are induced by d-galUA in R. toruloides.

**FIG 6:**
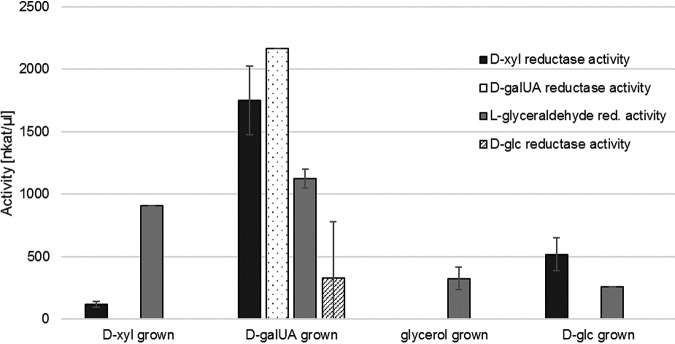
Reductase assays with whole-cell lysates of R. toruloides IFO0880 pregrown on different carbon sources. Measurements were performed from biological triplicate cultures. Error bars represent SD.

In light of the previous observations, particularly regarding growth physiology and enzymatic characterizations, we aimed to investigate which substrates are able to induce monosaccharide reductase activity in R. toruloides *in vivo*. To this end, reductase assays were performed with whole-cell lysates following growth for 24 h on either d-xyl, d-galUA, d-glc, or glycerol. The enzymatic activity of the cell lysates was tested using d-xyl, d-galUA, d-glc, and l-glyceraldehyde as substrates by measuring the NADPH concentration loss over time. All lysates showed l-glyceraldehyde reductase activity, albeit to various extents (Fig. 6). In contrast, d-galUA and d-glc reductase activities were specific for the cultures grown on d-galUA. Intriguingly, while d-xyl reductase activity was somewhat less specifically induced, growth on d-galUA clearly led to the strongest induction. Considering that RTO4_9774 is induced about 5- to 7-fold on d-galUA over glycerol and d-glc, respectively (Tables 1 and 2), these results suggest that a major part of the observed d-xyl reductase activities is contributed by RTO4_9774 functioning as pentose reductase. The same might be true for the low d-glc reductase activity recorded specifically after induction on d-galUA. In addition, these activities could help to explain the accelerated d-xyl and d-glc consumption in the presence of d-galUA as seen in the mixed cultures (Fig. 1).

### Identification of RTO4_13270 as the first basidiomycete transcription factor involved in the catabolism of d-galUA.

**FIG 7:**
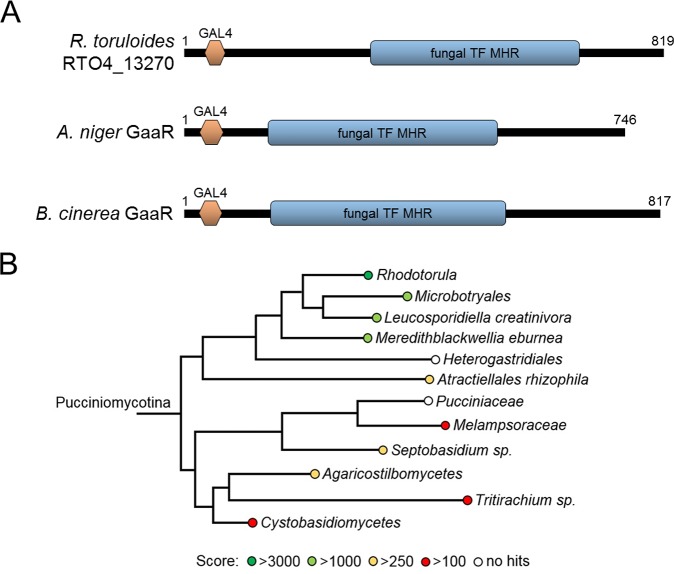
(A) Domain analysis of putative d-galUA transcription factors show presence of GAL4 and fungal transcription factor middle homology region (TF MDH) domains; however, RTO4_13270 has low overall homology to the known ascomycete GaaRs. (B) Phylogenetic tree of putative RTO4_13270 orthologs. Among the Pucciniomycotina subdivision, proteins most closely related to RTO4_13270 are found in the genera *Leucosporidiella* and *Meredithblackwellia*, and Microbotryales. To visualize sequence relationships, BLAST scores were color-coded to the fungal family/genus/species according to their homology. Families with an empty circle did not return any result (these sequences were not publicly available on the Joint Genome Institute’s server).

A putative transcription factor, RTO4_13270, exhibited induction on d-galUA (Tables 1 and 2). Moreover, disruption of this gene by T-DNA insertion resulted in a very large fitness disadvantage for growth in d-galUA (Table 2 and Fig. 4), indicating that it might be a key regulator for the catabolism of this carbon source. This novel TF belongs to the same Gal4-like family as the known d-galUA-responsive TF from ascomycetes, GaaR (30, 31), but is otherwise phylogenetically unrelated (Fig. 7A). We assessed conservation of RTO4_13270 in basidiomycetes by searching for homologs in the fungal proteomes from the Pucciniomycotina (to which R. toruloides belongs [Fig. 7B]). Overall, RTO4_13270 was found to be highly conserved in the *Rhodotorula/Rhodosporidium* genus, still relatively conserved in closely related groups, but not at all conserved beyond the Pucciniomycotina.

## DISCUSSION

It has been shown that the red yeast R. toruloides exhibits strong growth on pectin-derived monosaccharides, including d-galUA, d-xyl, l-ara, and d-glc (18). Red yeasts likely fill an opportunistic niche on pectic substrates and assimilate the monosaccharides liberated by the enzymes of other microorganisms (32). For example, *Rhodotorula* species were found to colonize grapes in the presence of other pectinolytic fungi potentially releasing sugars from the fruit tissue (33). In this study, we characterized the d-galUA utilization pathway of R. toruloides by a combination of transcriptomics, genomewide fitness profiling, and biochemical analysis of purified enzymes.

**FIG 8:**
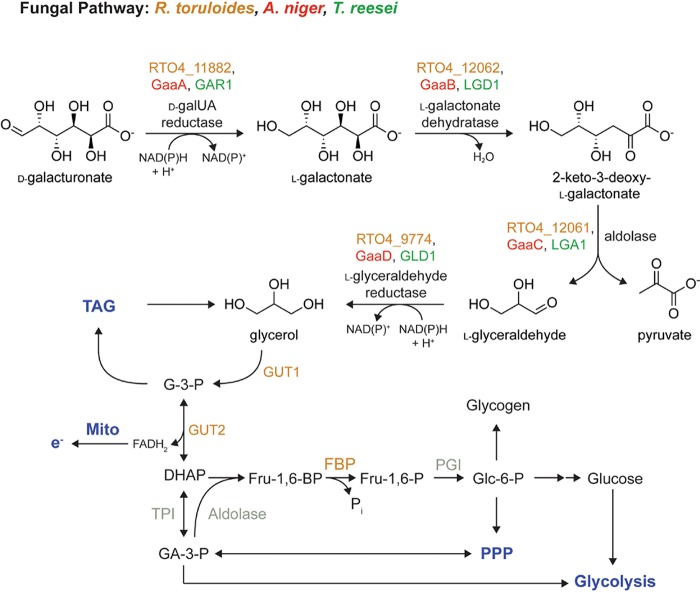
Model of R. toruloides d-galUA catabolism based on combined RNA-seq and RB-TDNA-seq analysis. While the initial enzymatic steps follow the same strategy as known from the ascomycete pathway, genomewide transcriptional and fitness profiling revealed an expanded role for glycerol metabolism and gluconeogenesis in d-galUA catabolism. TAG, triacylglycerol; Mito, mitochondria; PPP, pentose phosphate pathway.

R. toruloides utilizes a nonphosphorylative d-galUA catabolic pathway, as observed in ascomycete filamentous fungi (Fig. 8) (9). The R. toruloides pathway is similar to the T. reesei pathway compared to the A. niger pathway due to the absence of a GaaA homolog and the presence of a functional GAR1 homolog (Fig. 5) (34). The conserved enzymes are highly induced by d-galUA, required for fitness and shown to have the predicted biochemical activities for each catabolic step *in vitro*. When comparing the catalytic activities to those reported for T. reesei and A. niger, the substrate affinities (*K_m_* values) of the R. toruloides enzymes are for the most part surprisingly similar (8, 10, 11, 35) (Table S3). However, particularly for the dehydratase RTO4_12062, the aldolase RTO4_12061, and the l-glyceraldehyde reductase RTO4_9774, the maximal velocities are about 4 to 500 times higher, suggesting that this might contribute to the high efficiency of the pathway flux. Interestingly, the dehydratase and aldolase are clustered in the genome, whereas the pathway reductases are found elsewhere in the genome. Even though this genomic arrangement differs from the state in the filamentous ascomycetes, in which the aldolase gene (*gaaC*) forms a gene pair with the initial d-galUA reductase gene (*gaaA*) (9), it may indicate that a tight coregulation of the enzyme expression (via a shared promoter region) is beneficial for the pathway as a whole and for the aldolase in particular.

10.1128/mSystems.00389-19.7 TABLE S3 Comparison of catalytic activities of conserved enzymes in the nonphosphorylative d-galUA catabolic pathway between R. toruloides, T. reesei, and A. niger. Download Table S3, PDF file, 0.04 MB. Copyright © 2019 Protzko et al. 2019 Protzko et al. This content is distributed under the terms of the Creative Commons Attribution 4.0 International license.

Another intriguing observation of our study was that the d-galUA catabolism led to an enhanced coutilization of d-glc and d-xyl, which are the most abundant hexose and pentose sugars in plant biomass and therefore a primary target of biorefinery concepts. This is notable, since it was shown that the presence of d-galUA (at low pH) inhibits the assimilation of d-xyl, d-gal, and l-ara in the commonly used fermentation host Saccharomyces cerevisiae, possibly via competitive inhibition of the main transporter Gal2p and a general weak acid toxicity (36). Even though the pH used in this study was higher, the high efficiency of catabolism of d-galUA in R. toruloides may allow sufficient ATP to overcome intracellular toxicity of pathway intermediates or proton efflux, whereas S. cerevisiae is incapable of d-galUA assimilation without engineering (37–39).

Coconsumption of d-galUA with d-glc and d-xyl might furthermore benefit from the multifunctional role of RTO4_9774, as mentioned above. RTO4_9774 is induced on d-galUA, and its additional activities as a pentose reductase and d-glc reductase will help to assimilate these sugars under mixed-culture conditions. Competition of sugars for available enzymes may explain the delay in d-galUA consumption, and the induction of additional catabolizing enzyme genes by d-galUA, as visible in RNA-seq and in the reductase assay (Fig. 6), could further explain the higher growth rate in mixed-sugar cultures. Additionally, the catabolism pathways of d-glc and d-galUA have little overlap and could allow parallel utilization, and d-galUA catabolism does not appear to be repressed by physiological systems such as catabolite repression in the presence of high d-glc concentrations. Future experiments with the single gene deletions will help to address this matter. Excitingly, the observation of sugar coconsumption at high sugar loadings is of high biotechnological relevance for efficient mixed-sugar fermentations of pectin-rich biomass.

The contribution of a specific d-galUA uptake system in R. toruloides to high flux and low competition with other sugars can be potentially attributed to MFS-type transporters. One MFS-type sugar transporter, RTO4_9841 (class 2.A.1.1 [http://www.tcdb.org]), exhibited strong induction on d-galUA (mean fragments per kilobase per million [FPKM] of 4,605), and disruption of this gene resulted in a significant fitness defect. Interestingly, its close homolog, RTO4_9846, which probably resulted from a recent duplication event, is also induced on d-galUA, but it did not cause a significant fitness defect and might therefore represent a pseudogene (see Materials and Methods). Surprisingly, phylogenetic analysis and comparison with the better-described MFS transporters of class 2.A.1.1 from N. crassa (Fig. S4) show that these transporters have higher sequence homology to annotated arabinose transporters (e.g., LAT-1 [40]) than to the GAT-1 family of d-galUA transporters found in ascomycetes. However, the relation of RTO4_9841 and 9846 to pentose transporters and their function in d-galUA uptake are currently unclear.

10.1128/mSystems.00389-19.4 FIG S4 MFS transporter phylogeny using the predicted MFS-type transporters from TCDB class 2.A.1.1 (sugar porter family) and those from the reference organism Neurospora crassa to help in the identification of putative substrates. Download FIG S4, PDF file, 0.3 MB. Copyright © 2019 Protzko et al. 2019 Protzko et al. This content is distributed under the terms of the Creative Commons Attribution 4.0 International license.

The combination of transcriptomics and functional genomics analysis identified downstream components highly relevant for high d-galUA catabolism. Moreover, our emerging model of d-galUA metabolism in R. toruloides may also serve as a road map for the engineering of d-galUA pathways in other organisms. In particular, three genes seem to be of high importance and received low fitness scores when disrupted by T-DNA insertions: *GUT1*, *GUT2*, and *FBP1*. The first two genes encode the enzymes glycerol kinase and mitochondrial glycerol 3-phosphate dehydrogenase, which are involved in the canonical glycerol metabolism pathway, as known from S. cerevisiae and filamentous fungi (41–43). An efficient glycerol metabolism therefore appears to be crucial for d-galUA assimilation. Moreover, since R. toruloides as an oleaginous yeast has highly efficient TAG biosynthesis and turnover, which is linked to glycerol metabolism at the stage of glycerol-3-phosphate (G-3-P), the d-galUA metabolism might hitchhike on these capacities and benefit from the high possible fluxes (19, 44). In addition, the FAD-dependent oxidation of G-3-P to dihydroxyacetone phosphate (DHAP) in the mitochondrial outer membrane by GUT2 may provide reducing power necessary for the conversion of relatively oxidized d-galUA. This might be supported by the participation of GUT2 in the G-3-P shuttle, which is involved in the maintenance of the NAD:NADH redox balance, for example, in S. cerevisiae (45). The necessity of the fructose bisphosphatase (FBP1) for d-galUA utilization may suggest involvement of gluconeogenesis. Further support for this hypothesis might be derived from the essentiality of the SNF1 complex (all three subunits) for growth fitness on d-galUA. This complex is highly conserved from yeast to humans, is an antagonist of carbon catabolite repression, and promotes gluconeogenesis in the absence of d-glc (23). A proper metabolic switch from d-glc to alternative carbon sources such as d-galUA, including activation of gluconeogenesis, is thus a clear prerequisite for efficient growth under this condition. It remains to be shown whether the newly identified TF RTO4_13270 is a target of the SNF1 complex, since one of its main functions is to activate several TFs by phosphorylation in yeast (46, 47). A downstream product of FBP1, glucose-6-phosphate, also represents an entry gate into the pentose phosphate pathway (PPP), which may be an additional way to generate the necessary reducing equivalents to redox balance the assimilation of d-galUA, an oxidized sugar acid substrate.

The characterization of the d-galUA catabolic pathway described in this work sets the basis for use of R. toruloides as a potential host for pectin-rich waste conversions. The novel enzymes and transporters described here may furthermore be valuable for biotechnological use in anaerobic microbes, such as S. cerevisiae. The present study also demonstrated that the molecular tools now available for R. toruloides make it an ideal model for the elucidation of basic biological concepts, such as carbon sensing, signaling, and substrate utilization (among many others), in basidiomycete fungi.

## MATERIALS AND METHODS

### Strains.

We used R. toruloides IFO0880 (also designated NBRC 0880), which was obtained from the NITE Biological Resource Center (https://www.nite.go.jp/nbrc/), for the growth assays, transcriptional analysis, and functional genomic analysis performed in this study.

### Culture conditions.

Unless otherwise stated, R. toruloides IFO0880 cultures were grown at 30°C in 50 ml of liquid medium in 250-ml baffled flasks with agitation at 250 rpm on a shaker. For strain maintenance rich medium conditions, yeast peptone dextrose (YPD) medium was used supplemented with 2% (wt/vol) d-glc. For growth assays, IFO0880 was precultured in 0.68% (wt/vol) yeast nitrogen base (YNB) without amino acids (Sigma-Aldrich; Y0626), pH 5.5, with 2% (wt/vol) d-glc for 4 days. Afterwards, cells were washed in YNB without carbon. YNB cultures supplemented with the respective carbon source and 0.2% (wt/vol) ammonium sulfate were inoculated with an initial OD at 600 nm (OD_600_) of 0.1. For mixed cultures, substrates were combined in equal amounts to the respective final concentration.

To investigate the growth of R. toruloides on different carbon sources, we tested 50 mM d-glc, d-galUA, and d-xyl either as single carbon sources or in combination. Furthermore, growth on d-glc and d-galUA was also tested at a higher concentration, 500 mM. Assays performed with a 50 mM substrate concentration were performed using sterile 96-well plates with cover. The plates were constantly shaken at 1,000 rpm at 30°C in a thermoblock. For assays with a 500 mM substrate concentration, cells were cultured in a 50-ml volume using 250-ml baffled shake flasks. For transcriptional analysis, R. toruloides was grown in media containing either 50 mM d-galUA or glycerol. E. coli strains were cultured in LB medium supplemented with the respective antibiotics ampicillin (100 μg/ml) and chloramphenicol (68 μg/ml) and incubated at 37°C and with constant agitation at 250 rpm.

### Sugar consumption assays.

At each time point of absorbance measurement, aliquots were taken and diluted with water to a concentration of 1:100. The samples were centrifuged at for 4 min at 15,000 × *g* and 4°C. Subsequently, 400 μl of the supernatant was transferred into a new Eppendorf tube. To determine the remaining sugar content, we used high-pH anion exchange chromatography (HPAEC). Prior to measuring, aliquots of the supernatants were further diluted to a final concentration of 1:5,000. Quantification of monosaccharides was performed as described in references 7 and 48. For elution of neutral monosaccharides, samples were injected into a 3- by 150-mm CarboPac PA20 column at 30°C by using an isocratic mobile phase of 10 mM NaOH and a flow rate of 0.4 ml/min over 13 min.

### RNA sequencing and analysis.

An overnight starting culture of R. toruloides IFO0880 was diluted to an OD (at 600 nm) of 0.2 in 100 ml of YPD (BD Biosciences, San Jose, CA; BD242820) in a 250-ml baffled flask and incubated 8 h at 30°C and 200 rpm on a platform shaker. In this time the culture reached an OD of 1.0. Cultures were then pelleted by centrifugation at a relative centrifugal force (RCF) of 3,000 at room temperature (RT) for 5 min and washed twice with YNB (BD Biosciences; BD291940) medium without a carbon source. This starter culture was then used to inoculate triplicate cultures in YNB plus 2% d-glc (Sigma-Aldrich, St. Louis, MO; G7528), 2% d-galUA, and 2% glycerol (Sigma-Aldrich; G5516) at an OD of 0.1 in 100-ml cultures in 250-ml baffled flasks. Growth on each carbon source was then allowed to proceed to the onset of stationary phase at an OD of 2.0. Approximately 10 OD units were then pelleted and frozen at –80°C for DNA extraction and analysis. Total RNA was isolated using an RNeasy minikit (Qiagen; catalog no. 74104) using on-column DNA digestion (Qiagen; catalog no. 79254). RNA-seq libraries were sequenced on an Illumina HiSeq 4000 system at the QB3 Vincent J. Coates Genomic Sequencing Laboratory (http://qb3.berkeley.edu/gsl/) using standard mRNA enrichment, library construction, and sequencing protocols. Approximately 40 million 50-bp single-end reads were acquired per replicate per condition. Transcript abundances and differential expression were calculated with HiSat 2.1.0, StringTie 1.3.3b, and Ballgown 2.8.4 (49) by mapping against R. toruloides IFO0880 v4 reference transcripts (https://genome.jgi.doe.gov/Rhoto_IFO0880_4/Rhoto_IFO0880_4.home.html).

To cluster genes with significant differential expression, genes with adjusted *P* values of <0.05 across all three conditions were filtered to remove genes with low expression (FPKM < 5 under all conditions) and small fold changes (<2-fold). FPKM values were then clustered using Pearson correlation as the similarity metric and average linkage as the clustering method (hclust function R).

### Barcode sequencing and fitness analysis.

Fitness analysis of a pooled, barcoded R. toruloides T-DNA mutant library was performed as described in reference 22, with minor alterations. Briefly, three aliquots of the previously generated pool of random Agrobacterium tumefaciens insertional mutants were thawed on ice and then inoculated at an OD (600 nm) of 0.2 in 100 ml YPD (BD Biosciences, San Jose, CA; BD242820) in a 250-ml baffled flask and incubated 8 h at 30°C and 200 rpm on a platform shaker. In this time the mutant pools reached an OD of 0.8. At this time, a time zero reference sample of the starter culture was collected for all three replicates. Cultures were then pelleted by centrifugation at an RCF of 3,000 and room temperature for 5 min and washed twice with YNB (BD Biosciences; BD291940) medium without a carbon source. Each starter culture was then used to inoculate new cultures in YNB plus 2% d-glc (Sigma-Aldrich, St. Louis, MO; G7528), 0.2% d-glc, 2% d-galUA, 0.2% d-galUA, and 2% glycerol (Sigma-Aldrich; G5516) at an OD of 0.1 in 100-ml cultures in 250-ml baffled flasks. Growth on each carbon source was then allowed to proceed to the onset of stationary phase (14, 14, 36, 19, and 64 h, respectively, with ODs at sampling of 5, 1.3, 4.1, 0.8, and 1.9, respectively). Approximately 10 OD units were then pelleted and frozen at –80°C for DNA extraction and analysis. Barcode amplification, sequencing, and fitness analysis were performed as described in reference 22. Briefly, barcode sequences are amplified in a PCR that produces Illumina sequencing-ready amplicons. Occurrences of each barcode sequence are then counted in each sample. The locations of hundreds of thousands of barcodes of these are known because the pooled mutant library has been previously deep sequenced and unique barcodes have been mapped to their respective positions on the genome. For each of these mapped barcodes, we compute a strain fitness score: a log_2_ ratio of counts in the experimental sample versus the time zero reference sample. A strongly negative fitness score indicates slow growth for the strain bearing that insertion relative to the general population of the mutant pool. To control for differences in sequencing depth between samples, fitness scores are normalized such that the average fitness score in any condition is zero, as it is generally observed that most T-DNA insertions do not cause a significant phenotype under any one condition. To compensate for potential background mutations in any particular barcoded strain, these strain fitness scores are aggregated among all insertions disrupting the same gene by taking a weighted harmonic mean of all scores, with barcodes weighted in proportion to their relative sequencing depths. Note that these fitness scores are not scaled to the number of generations that occurred in a given experiment, so they cannot be interpreted in terms of absolute growth rates under a given condition without additional information. To assess statistical significance of these gene fitness scores, a modified T-statistic is then calculated, which encapsulates consistency scores both between biological replicates and between barcodes (22).

### Lysate assay.

Cells were precultured for 2 days in 3 ml of YNB (pH 5.5) plus 2 % d-glc in 24-well deep-well plates at 30°C with agitation at 700 rpm. Cells were washed 3 times in 1 ml of YNB medium without a carbon source, followed by 24 h of induction in 3 ml of YNB with the respective carbon source. A volume of 1 ml of the culture was centrifuged at 3,500 rpm and 4°C for 2 min to lyse the cells. The cell pellet was disrupted in 400 μl of lysis buffer (50 mM NaOH, 1 mM EDTA, 1% Triton X-100) with 0.5-mm glass beads using a laboratory bead mill (BeadBug microtube homogenizer; SLG Gauting) for 1 min at maximum speed and RT. For the determination of reductase activity by measuring the loss of NADPH concentration over time, the complete supernatant after cell lysis was used. The total reaction mixture of 100 μl contained 50 mM substrate (d-xyl, d-glc, d-galUA, or glycerol), 0.3 mM NADPH, 100 mM sodium phosphate buffer (pH 7), 0.1 μl of Tween 20, and 50 μl of the different cell lysates. The assay was performed at 30°C in UV-compatible 96-well plates (Corning, Germany) in an Infinite M200 PRO reader (Tecan, Germany) for 5 min in total, measuring the optical density at 340 nm every 15 s. Prior to each measurement, the plates were shaken for 5 s.

### *In vitro* activity assays.

For determination of the activities of *in vitro*-purified enzymes, RTO4_11882, RTO4_12062, RTO4_12061, and RTO4_9774 were cloned into a custom-made HIS6-Tobacco Etch Virus (TEV) expression vector under the control of a T7 promoter and transformed into E. coli (Rosetta) cells. The resulting N-terminally tagged proteins contained an in-frame HIS6-TEV (MGHHHHHHDYDIPTTENLYFQG) fusion sequence. Protein expression was induced by using 1 mM isopropyl-β-d-thiogalactopyranoside (IPTG) at an OD_600_ of 0.4 to 0.6, and cells were incubated overnight at 16°C with agitation at 250 rpm before being harvested by centrifugation at 4°C. For lysis, cells were resuspended in lysis buffer (see above), 20 mg/ml of lysozyme was added, and the suspension was incubated for 1 h at 37°C rotating at 10 rpm. After 30 min and 45 min of incubation, 2.5 μl of DNase I was added. Cell debris was precipitated by centrifugation at 16,000 × *g* and 4°C for 20 min. The supernatant was used for protein purification by immobilized-metal affinity chromatography (IMAC) (50). Chosen elutions were desalted in Vivaspin columns (10,000-Da molecular weight [MW] cutoff; Sartorius, Germany) with storage buffer (20 mM sodium phosphate, 20 mM NaCl [pH 7]) by centrifugation at 3,000 × *g* and 4°C.

Since it was observed that the tagged l-galactonate dehydratase from Hypocrea jecorina (Trichoderma reesei) showed reduced activity (10), the affinity tag of the putative l-galactonate dehydratase was removed by incubation with an in-house-purified and His-tagged TEV protease (ratio, 40:1 [mg:mg]) overnight at 4°C. In a second IMAC, the tag-free protein was collected in the flowthrough and desalted as described above. Protein concentrations were determined with a Bradford assay using Roti-Quant reagent (Roth, Germany) and a bovine serum albumin (BSA) calibration series. The enzymatic activity of the two reductases was determined as described above, but with a total reaction volume of 200 μl and 1 to 2 μg of purified protein instead of cell lysates.

For RTO4_12061, a thiobarbiturate assay according to reference 51 was performed. A 200-μl reaction mixture (50 mM sodium phosphate buffer [pH 7.0], 60 mM pyruvate, and various l-glyceraldehyde concentrations or 25 mM l-glyceraldehyde and various pyruvate concentrations) was mixed with 3.3 μg of protein. Samples were removed during the linear range of the reaction. The reaction was stopped and developed as described, but with half the quantities.

For RTO4_12062, a modified semicarbazide assay was performed (29). One microgram of enzyme was mixed with different l-galactonate concentrations in 5 mM MgCl_2_ and 50 mM sodium phosphate (pH 7.0) in a 180-μl total volume. Within the linear range of the reaction, 80-μl samples were taken, mixed with 20 μl of 2 M HCl, vortexed, and centrifuged at 16,000 × *g* and 4°C for 10 min. Forty microliters of the supernatant was pipetted to 160 μl of semicarbazide solution (1% [wt/vol] semicarbazide, 1% [wt/vol] sodium acetate) and incubated at RT for 30 min. The absorbance was measured at 250 nm in UV-compatible 96-well plates (Corning, Germany). Linear l-galactonate was obtained by dissolving l-galactono-1,4-lactone (Sigma, Germany) in water and adding sodium hydroxide until the pH stopped changing and could be set to pH 7 with 1 M sodium phosphate.

### Synteny identification.

Protein sequences of GAR1, GAR2, LGD1, LGA1, and GLD1 from Trichoderma reesei were acquired from The Universal Protein Resource (UniProt; https://www.uniprot.org/) databases. Sequences were compared by BLASTp (https://blast.ncbi.nlm.nih.gov/Blast.cgi) with T. reesei sequences from FungiDB (http://fungidb.org/fungidb/), and the results illustrating 100% sequence identity were chosen for determination of scaffolds, strand orientation (+ or − strand), and genomic DNA sequence (from ATG to the stop codon). Aspergillus niger protein sequences for *gaaA*, *gaaB*, *gaaC*, and *gaaD* were acquired from FungiDB. The GAR2 sequence of T. reesei (from UniProt) was used to determine the A. niger ortholog in FungiDB by using BLASTp. Their strand orientation (+ or – strand), position on the chromosomes, and genomic DNA sequence (from ATG to the STOP codon) were determined in the FungiDB. For R. toruloides IFO0880, protein sequences of RTO4_11882, RTO4_12062, RTO4_12061, and RTO4_9774 were acquired from MycoCosm (http://jgi.doe.gov/fungi) using the R. toruloides IFO0880 v4 genome. Their strand orientation (+ or – strand), position on the scaffolds, and genomic DNA sequence (from ATG to the stop codon) were determined in MycoCosm. In general, the DNA sequence length of all genes was determined to create an approximation of the gene length in the respective figure. The amino acid sequence of transcription factor RTO4_13270 was retrieved from the Joint Genome Institute (JGI) database (https://genome.jgi.doe.gov/Rhoto_IFO0880_4/Rhoto_IFO0880_4.home.html) and compared to all entries in the Pucciniomycotina tree of MycoCosm by using BLASTp. The best hit of each subgroup was taken and its BLAST score was used to determine homology. The phylogeny of the Pucciniomycotina was taken from MycoCosm and modified with the results from the homology search.

### Phylogenetic analyses.

The protein sequences of RTO4_11882, RTO4_12062, RTO4_12061, and RTO4_9774 from IFO0880 v4 were retrieved from MycoCosm (http://jgi.doe.gov/fungi) and compared to those of their homologs in T. reesei and A. niger. Additionally, the conservation of these genes between R. toruloides and representative basidiomycete organisms was determined by phylogenetic analysis. Amino acid sequences of T. reesei and A. niger were retrieved from UniProt and FungiDB, respectively, as described in the previous section. The protein sequences of the basidiomycete organisms were retrieved from MycoCosm by running BLASTp of the R. toruloides genes against the Basidiomycota tree. Genes with high sequence similarity and from representative families were used for the following analyses. The phylogenetic tree was constructed using Clustal Omega (https://www.ebi.ac.uk/Tools/msa/clustalo/) (52–54). The design was finalized using FigTree v1.4.3 (http://tree.bio.ed.ac.uk/software/figtree/) to create a rectangular tree with increasing node ordering and branches transformed to cladogram.

Similarly, R. toruloides proteins annotated as sugar porters or MFS-type by Pfam (http://pfam.xfam.org/) (55–59) were analyzed using the Transporter Classification Database (TCDB; http://www.tcdb.org/) (60). Amino acid sequences of R. toruloides proteins classified as category 2.A.1.1 (sugar porter family) were retrieved from MycoCosm and compared to sequences of N. crassa OR74a transporter proteins of TCDB category 2.A.1.1 (60), which were gathered from FungiDB. For RTO4_9846, due to doubts about correct annotation, an alternative gene model was proposed and used for the phylogenetic analysis. The gene model can be found at https://mycocosm.jgi.doe.gov/cgi-bin/dispGeneModel?db=Rhoto_IFO0880_4&id=9846. As described above, a phylogenetic tree was created with Clustal Omega using these sequence data. The tree was finalized using FigTree v1.4.3 with a polar tree layout, increasing node ordering and with branches transformed to cladogram.

### Data availability.

RNAseq data are available at the NCBI Gene Expression Omnibus (GEO) under accession number GSE127536. BarSeq data are available at the NCBI Sequence Read Archive (SRA) under accession number PRJNA524012.
