# Supplementary figures and images for: Genomewide and Enzymatic Analysis Reveals Efficient d-Galacturonic Acid Metabolism in the Basidiomycete Yeast Rhodosporidium toruloides
Source: mSystems. 2019 Dec 17;4(6):e00389-19. doi: 10.1128/mSystems.00389-19 (PMC6918025; doi:10.1128/mSystems.00389-19)

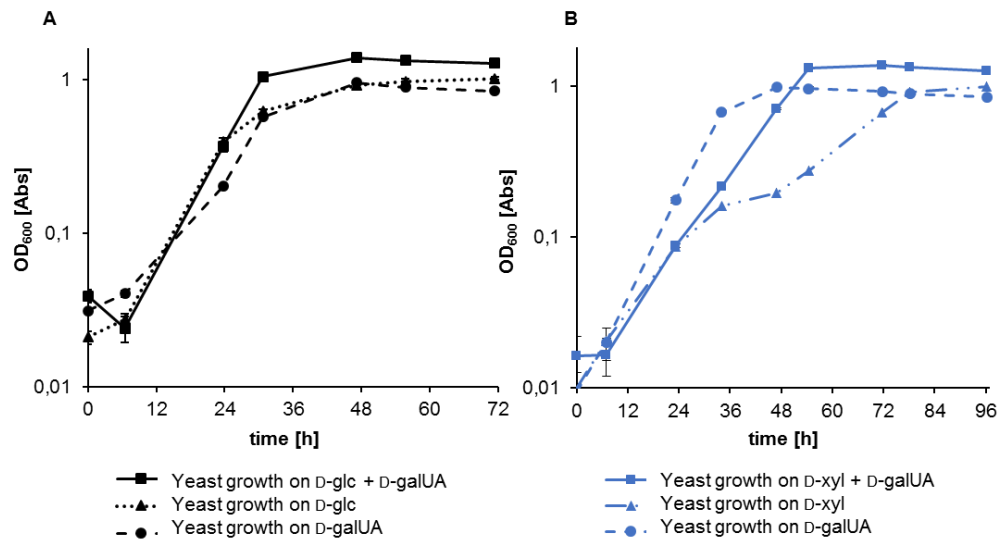

Supplement: FIG S1 [file mSystems.00389-19-sf001.pdf]

**A**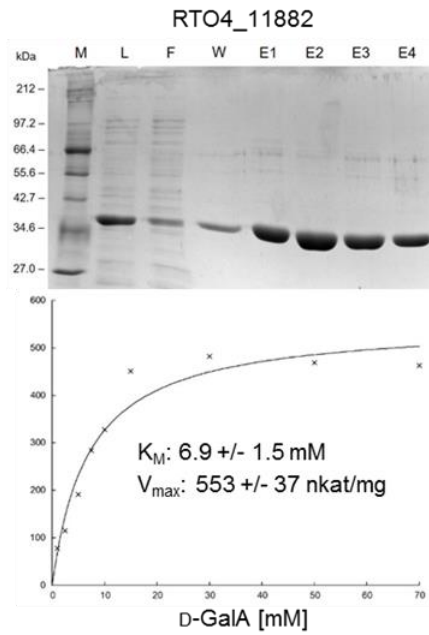**C**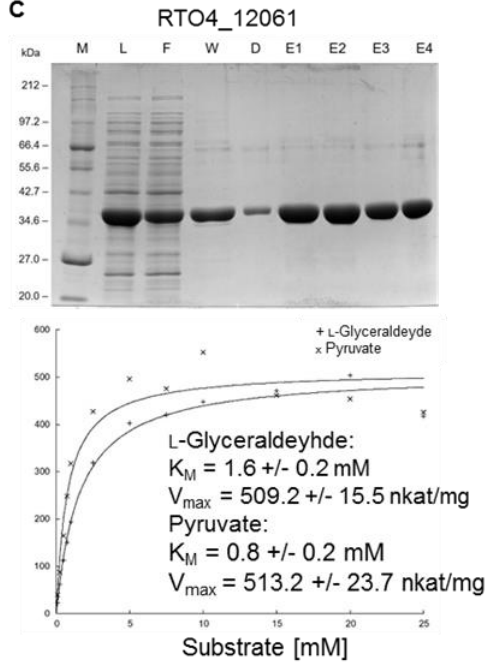**B**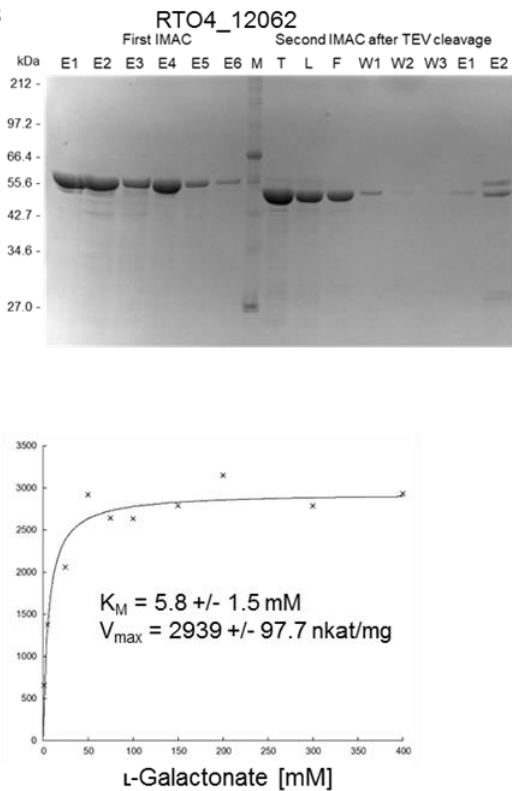**D**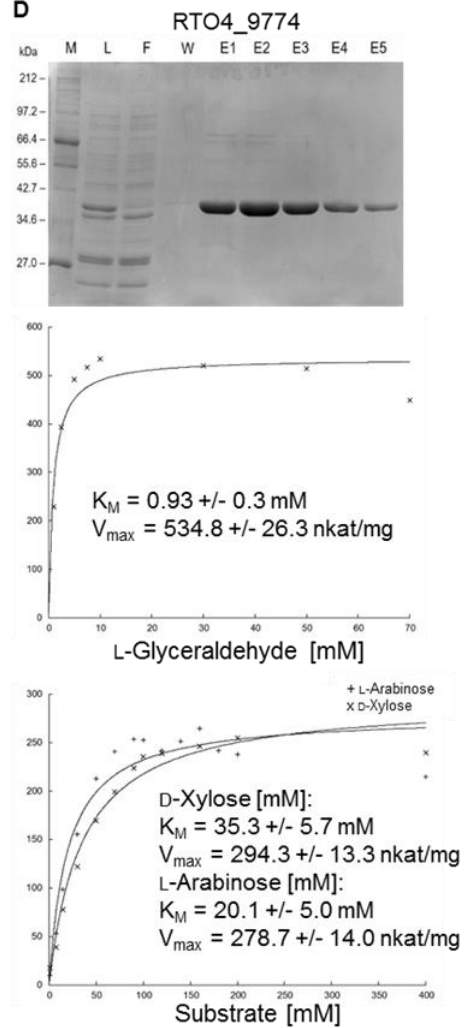

Supplement: FIG S2 [file mSystems.00389-19-sf002.pdf]

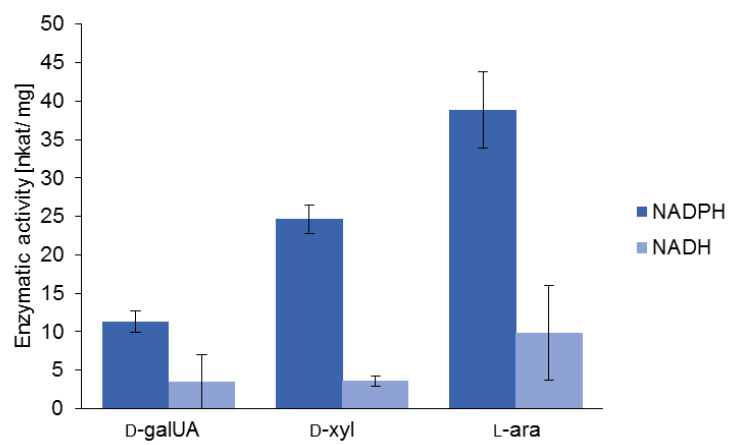

Supplement: FIG S3 [file mSystems.00389-19-sf003.pdf]

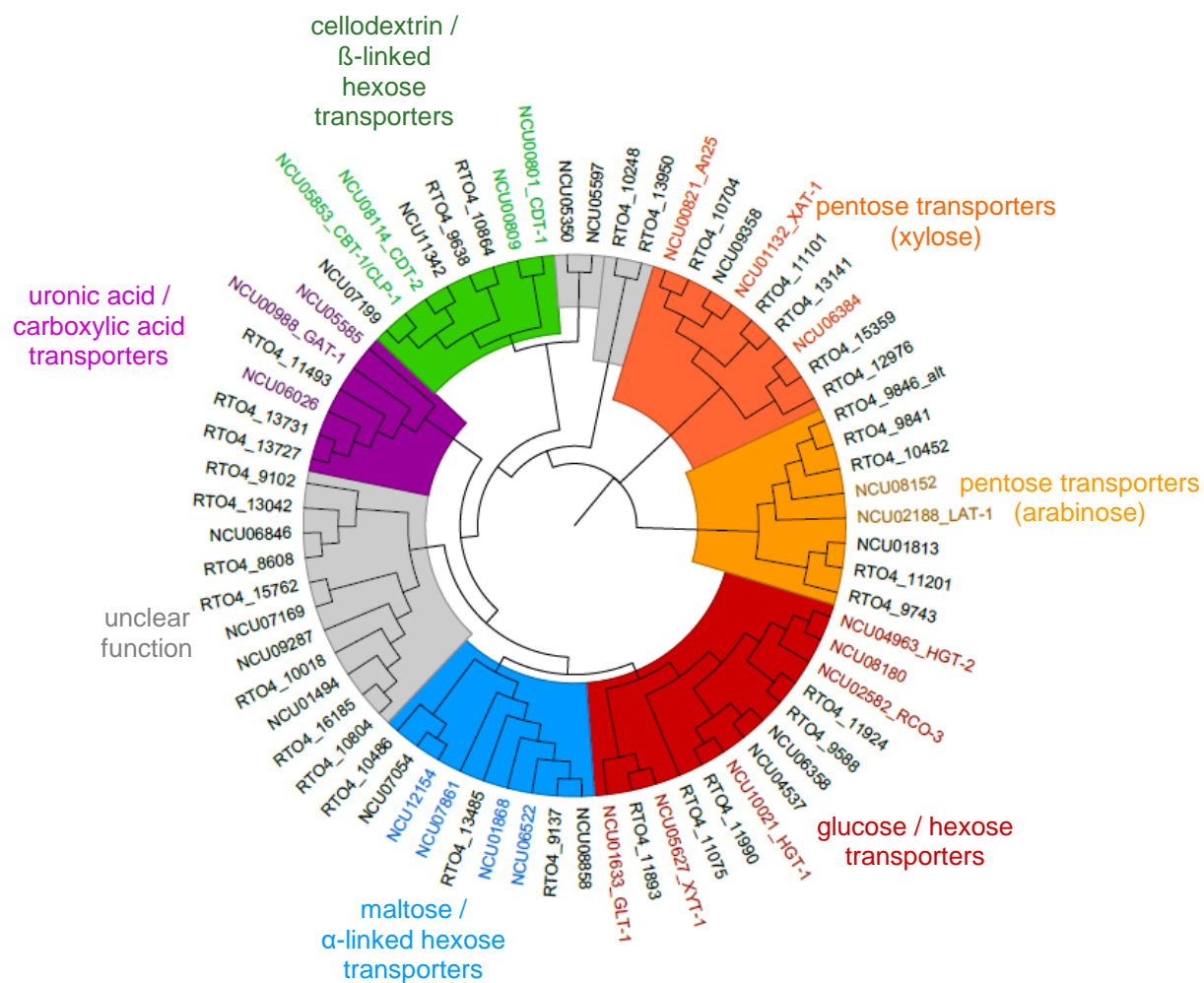

Supplement: FIG S4 [file mSystems.00389-19-sf004.pdf]
