# Supplementary material for: Genomewide and Enzymatic Analysis Reveals Efficient d-Galacturonic Acid Metabolism in the Basidiomycete Yeast Rhodosporidium toruloides
Source: mSystems. 2019 Dec 17;4(6):e00389-19. doi: 10.1128/mSystems.00389-19 (PMC6918025; doi:10.1128/mSystems.00389-19)
Supplement: TABLE S2 [file mSystems.00389-19-st002.pdf]

Protein ID	Name	Description	Mean FPKM			Fitness Score		
			D-galUA	gly	D-glc	D-galUA	gly	D-glc
**Glycerol Utilization**								
RTO4_15423	FBP1	Fructose-1,6-bisphosphatase I	852	142	161	-2.0	-0.8	0.0
RTO4_11039	GUT2	Glycerol-3-P dehydrogenase	405	31	49	-2.2	-3.1	-0.1
RTO4_11022	GUT1	Glycerol kinase	122	49	27	-3.2	-3.4	0.2
RTO4_10704	STL1	Glycerol transporter	2361	197	273	0.1	-0.6	-0.2
**Galactose Utilization**								
RTO4_13990	GAL1	Galactokinase	87	78	46	-0.9	0.0	0.3
RTO4_8969	GAL10	UDP-glucose 4-epimerase	490	440	241	N/A	N/A	N/A
RTO4_9959	PGM2	Phosphoglucomutase	202	225	82	0.0	0.0	0.2
RTO4_13711	PRM15	Phosphoglucomutase	27	22	20	0.2	0.3	0.3
**NADPH Production**								
RTO4_15167	GND1	6-phosphogluconate dehydrogenase	2328	1187	894	N/A	N/A	N/A
RTO4_10000	ZWF1	Glucose-6-phosphate 1-dehydrogenase	600	311	173	N/A	N/A	N/A
RTO4_12761	MAE1	Malic enzyme	86	492	121	0.1	0.2	-0.1
**Regulators of Glucose Metabolism**								
RTO4_10259	RIM11	Glycogen synthase kinase	431	775	334	-3.3	-2.3	0.1
RTO4_14809	SNF4	AMP-activated protein kinase subunit	18	17	14	-3.5	-1.7	-0.6
RTO4_15357	UBP14	Ubiquitin carboxyl-terminal hydrolase	30	20	26	-1.0	0.3	0.0
RTO4_16778	SNF1	Carbon catabolite-derepressing protein kinase	54	69	53	-3.5	-1.7	0.0
RTO4_8382	SIP2	AMP-activated protein kinase subunit	25	44	30	-2.2	-0.3	0.5

Protein ID	Name	Description	Mean FPKM			Fitness Score		
			D-galUA	gly	D-glc	D-galUA	gly	D-glc
**G proteins**								
RTO4_15484	CDC42	Rho-like GTPase	455	576	370	-2.0	-2.8	0.6
RTO4_14899	SEC2	Rab guanine exchange factor	29	25	19	-2.0	-1.0	0.0
RTO4_15198	RAB6A	Ras-related GTPase	405	369	256	-1.2	-0.3	-0.5
RTO4_16107	RIC1	RAB6A-GEF complex protein	26	35	29	-1.1	-0.7	-0.5
RTO4_14622		RAB6A-GEF complex protein	24	20	18	-1.3	-0.4	-0.4
**tRNA Thiolation**								
RTO4_10764	NCS2	tRNA 2-thiolation protein 2	21	11	17	-0.8	-1.2	0.3
RTO4_11341	UBA4	Adenylyltransferase and sulfurtransferase	15	9	17	-0.5	-0.8	0.5
RTO4_12817	NCS6	tRNA 2-thiolation protein 1	44	39	56	-0.5	-0.9	0.4
RTO4_14716	IKI3	Elongator complex protein 1	20	18	23	-0.4	-0.7	0.2
**Miscellaneous genes required for growth on D-galUA and glycerol**								
RTO4_16598	LARGE	Glycosyltransferase-like protein	42	57	67	-1.2	1.2	0.3
RTO4_9368		Similar to glycosyltransferases	38	38	45	-1.1	0.4	0.4
RTO4_9490		Similar to glycoside hydrolase family 28 proteins	22	31	35	-1.4	2.0	0.3
RTO4_14174		Pentatricopeptide repeat protein	70	72	152	-1.1	0.1	0.1
RTO4_13697	MCM4	DNA replication licensing factor MCM4	40	106	55	-1.0	-0.7	0.0
RTO4_12505		Hypothetical protein	25	45	32	-1.2	-1.0	0.9
RTO4_12325	SIT1	Ferrioxamine B transporter	124	2	457	-1.0	-0.2	-0.1
RTO4_14610	BAT1	Branched-chain amino acid aminotransferase	431	286	645	-2.8	-3.5	0.0
RTO4_16027		Acetylornithine transaminase	4	4	4	-2.0	-3.1	-0.5
RTO4_13539	OPT1	Oligopeptide transporter	206	444	196	-1.0	-2.1	-0.3
RTO4_8402		Proteophosphoglycan	26	23	33	-3.0	-3.3	-1.1
