# Supplementary material for: Genomewide and Enzymatic Analysis Reveals Efficient d-Galacturonic Acid Metabolism in the Basidiomycete Yeast Rhodosporidium toruloides
Source: mSystems. 2019 Dec 17;4(6):e00389-19. doi: 10.1128/mSystems.00389-19 (PMC6918025; doi:10.1128/mSystems.00389-19)
Supplement: TABLE S3 [file mSystems.00389-19-st003.pdf]

Protein	Substrate(s)	Activity<br>***R. toruloides***	Activity<br>***T. reesei***	Activity<br>***A. niger***	Ref.
D-Galacturonate reductase	D-galUA;<br>NADPH K_M<br>V_{Max}	**RTO4_11882**<br>6.9 ± 1.6 mM<br>553 ± 37 nkat/mg	**GAR1**<br>6 mM<br>666.8 nkat/mg	**GaaA**<br>0.175 mM<br>145 nkat/mg	(1, 2, this study)
L-Galactonate dehydratase	L-galactonate<br>K_M<br>V_{Max}	**RTO4_12062**<br>5.8 mM<br>2939 ± 97 nkat/mg	**LGD1**<br>5 mM<br>- (cell lysate)	**GaaB**<br>3.4 mM<br>5.6 nkat/mg	(1, 2, this study)
3-Deoxy-L-threo-hex-2-ulosonate aldolase	pyruvate<br>K_M<br>V_{Max}	**RTO4_12061**<br>0.8 ± 0.2 mM<br>513 ± 24 nkat/mg	**LGA1**<br>0.5 mM<br>108.4 nkat/mg	**GaaC**<br>-<br>-	(3, this study)
	L-glycerald.<br>K_M<br>V_{Max}	**RTO4_12061**<br>1.6 ± 0.1 mM<br>509 ± 16 nkat/mg	**LGA1**<br>1.2 mM<br>108.4 nkat/mg	**GaaC**<br>-<br>-	(3, this study)
L-Glycer-aldehyde reductase	L-glycerald.<br>K_M<br>V_{Max}	**RTO4_9774**<br>0.9 ± 0.3 mM<br>535 ± 26 nkat/mg	**GLD1**<br>0.9 mM<br>140 nkat/mg	**GaaD**<br>-<br>-	(4, this study)
	L-arabinose<br>K_M<br>V_{Max}	**RTO4_9774**<br>20.1 mM ± 5.0 mM<br>279 ± 14 nkat/mg	**GLD1**<br>-<br>-	**LarA**<br>54 ± 6 mM<br>500.1 nkat/mg	(5, this study)
	D-xylose<br>K_M<br>V_{Max}	**RTO4_9774**<br>35.3 ± 5.7 mM<br>294 ± 13 nkat/mg	**GLD1**<br>334 mM<br>450 nkat/mg	**LarA**<br>155 ± 15 mM<br>516.8 nkat/mg	(4, 5, this study)

Supplementary References

1. Martens-Uzunova ES, Schaap PJ. 2008. An evolutionary conserved d-galacturonic acid metabolic pathway operates across filamentous fungi capable of pectin degradation. Fungal Genet Biol 45:1449–1457. doi:10.1016/j.fgb.2008.08.002.
2. Kuorelahti S, Kalkkinen N, Penttilä M, Londesborough J, Richard P. 2005. Identification in the mold Hypocrea jecorina of the first fungal D-galacturonic acid reductase. Biochemistry 44:11234–11240. doi:10.1021/bi050792f.
3. Hilditch S, Berghäll S, Kalkkinen N, Penttilä M, Richard P. 2007. The missing link in the fungal D-galacturonate pathway: identification of the L-threo-3-deoxy-hexulosonate aldolase. J Biol Chem 282:26195–26201. doi:10.1074/jbc.M704401200.
4. Hackhofer M. 2017. Molecular and biochemical characterization of the pectionolytic capabilities of two basidiomycete red yeasts: *Rhodotorula mucilagionosa* and *Rhodosporidium toruloides*. Master’s thesis.
5. Mojzita D, Wiebe M, Hilditch S, Boer H, Penttilä M, Richard P. 2010. Metabolic engineering of fungal strains for conversion of D-galacturonate to meso-galactarate. Appl Environ Microbiol 76:169–175. doi:10.1128/AEM.02273-09.
